# The clinical utility of rapid exome sequencing in a consanguineous population

**DOI:** 10.1186/s13073-023-01192-5

**Published:** 2023-06-21

**Authors:** Dorota Monies, Ewa Goljan, Rapid Exome Consortium, Abdulaziz Mohammed Binmanee, Abdullah Ali Zafir Alashwal, Abdullah Mohammed Alsonbul, Abdulrahman A. Alhussaini, Alahmari Ali Abdallah, Ali Hussain Albenmousa, Ali Ibrahim Almehaidib, Ali Syed Akhtarul Hassan, Amal Salman Alseraihy Alharbi, Amro Alhabib, Antonello Podda, Badr Alsaleem, Bandar Bin Khalid Al Saud, Bassam Saleh Bin Abbas, Eissa Ali Faqeih, Fahad Badei Aljofan, Fahad Naser Alhazzani, Fouzah Awadh Alrowaily, Hamad Ibrahim Alzaidan, Hamoud Abdulkareem Almousa, Hawazen Saleh Alsaedi, Ibrahim Abdulaziz Ghemlas, Khalid Abdulrahman Alsaleem, Mahasen Saleh, Malak Alghamdi, Marwa Shams, Moath Alabdulsalam, Mohamed Salaheldin Bayoumy, Mohammad Ali Shagrani, Mohammed Abdulaziz Alowain, Mouhab Fakhreddine Ayas, Muhammad Qasim, Muneera J. Alshammari, Najeeb Shafat Qadi, Ohoud Saleh Alzahrani, Rand K. H. Arnaout, Reem Alhamad, Reem Walid Mohammed, Ruqaiah Saleh Altassan, Saad Ali Alghamdi, Saadiya Javed Khan, Saleh Abdulrahman Alalaiyan, Sameena Khan, Sultan Ibrahim Albuhairi, Talal Turki Algoufi, Tareq Mohammed Alayed, Tari Alofisan, Wajeeh Mohamed Aldekhail, Waleed Alhamoudi, Wesam Ibrahim Yousef Kurdi, Zuhair Abdalla Rahbeeni, Mirna Assoum, Muna Albreacan, Faisal Binhumaid, Shazia Subhani, Abdulmlik Boureggah, Mais Hashem, Firdous Abdulwahab, Omar Abuyousef, Mohamad H. Temsah, Fahad Alsohime, James Kelaher, Mohamed Abouelhoda, Brian F. Meyer, Fowzan S. Alkuraya

**Affiliations:** 1 Department of Clinical Genomics, Center for Genomic Medicine, King Faisal Specialist Hospital and Research Center, Riyadh, Saudi Arabia; 2 Department of Computational Science, Center for Genomic Medicine, King Faisal Specialist Hospital and Research Center, Riyadh, Saudi Arabia; 3 Department of Translational Genomics, Center for Genomic Medicine, King Faisal Specialist Hospital and Research Center, MBC-26, PO Box 3354, Riyadh, 11211 Saudi Arabia; 4 Department of Pediatrics, Pediatric Critical Care Unit, King Khalid University Hospital and College of Medicine, King Saud University, Riyadh, Saudi Arabia; 5 General Corporate Consultancy Department, King Faisal Specialist Hospital and Research Center, Riyadh, Saudi Arabia

**Keywords:** Rapid exome, Critical care, Novel gene-disease assertion, Reverse phenotyping

## Abstract

**Background:**

The clinical utility of exome sequencing is now well documented. Rapid exome sequencing (RES) is more resource-intensive than regular exome sequencing and is typically employed in specialized clinical settings wherein urgent molecular diagnosis is thought to influence acute management. Studies on the clinical utility of RES have been largely limited to outbred populations.

**Methods:**

Here, we describe our experience with rapid exome sequencing (RES) in a highly consanguineous population. Clinical settings included intensive care units, prenatal cases approaching the legal cutoff for termination, and urgent transplant decisions.

**Results:**

A positive molecular finding (a pathogenic or likely pathogenic variant that explains the phenotype) was observed in 80 of 189 cases (42%), while 15 (8%) and 94 (50%) received ambiguous (variant of uncertain significance (VUS)) and negative results, respectively. The consanguineous nature of the study population gave us an opportunity to observe highly unusual and severe phenotypic expressions of previously reported genes. Clinical utility was observed in nearly all (79/80) cases with positive molecular findings and included management decisions, prognostication, and reproductive counseling. Reproductive counseling is a particularly important utility in this population where the overwhelming majority (86%) of identified variants are autosomal recessive, which are more actionable in this regard than the de novo variants typically reported by RES elsewhere. Indeed, our cost-effectiveness analysis shows compelling cost savings in the study population.

**Conclusions:**

This work expands the diversity of environments in which RES has a demonstrable clinical utility.

**Supplementary Information:**

The online version contains supplementary material available at 10.1186/s13073-023-01192-5.

## Background

Monogenic (Mendelian) diseases are highly diverse disorders that can affect any organ and tend to be individually rare but collectively common. Thus, a typical clinician is very likely to encounter at least some of these diseases during their practice without the benefit of a prior clinical experience. This is particularly true in some settings that are enriched for monogenic diseases, e.g., neonatal and pediatric intensive care units [1]. Diagnosing these disorders was until recently a challenging endeavor that may take years, if at all successful. However, the advent of exome sequencing (ES) has upended this pattern by circumventing the prerequisite of an accurate clinical diagnosis, which historically posed a major bottleneck. Indeed, it is now possible to diagnose monogenic disorders even when the clinical diagnosis remains unrecognizable by the clinician [2].

Diagnosis of monogenic disorders molecularly, i.e., at the variant level, offers many advantages beyond the obvious benefit of having a definitive diagnostic label. Although the majority of monogenic disorders have no specific therapy, this is rapidly changing. In addition, the lack of specific therapy does not mean that important management choices cannot be informed by the molecular diagnosis, e.g., avoidance of futile or harmful interventions. The rare nature of most of these disorders underscores the value of molecular diagnosis for the purpose of building the necessary knowledgebase in regard to their natural history such that surveillance guidelines can be developed and data-driven prognostic information can be shared with patients and their families. Recurrence risk calculation can only be carried out accurately when a molecular diagnosis is obtained, and this is crucial for reproductive planning. These facets of clinical utility prompted ACMG (American College of Medical Genetics and Genomics) to endorse the adoption of ES as a first-tier test in patients with challenging forms of monogenic diseases such as multiple congenital malformations and developmental delay [3].

Rapid exome sequencing (RES) is a special type of ES wherein the entire pipeline from sample receiving to report generation is compressed to hours or days rather than weeks or months as is typical for routine clinical ES. The resource-intensive nature of RES is usually justified in select clinical scenarios in which a rapid molecular diagnosis is thought to inform acute clinical management decisions. Evidence of the latter has been generated incrementally since the initial application of rapid genome sequencing (RGS) in 2012. The pioneering anecdotes showing the value of RGS in intensive care units (neonatal and pediatric) quickly evolved into randomized case–control studies that clearly showed the clinical utility of this approach [1, 4, 5]. However, these studies were performed on outbred populations. Thus, there is a critical need to test the utility of RES in inbred populations where the genetic landscape can be distinct. We have previously described our preliminary experience with RES in our highly consanguineous population with a focus on diagnostic yield [2]. Here, we study a much larger cohort of 189 new cases and focus on the clinical utility of this approach.

## Methods

### Human subjects

All clinical teams were informed of the availability of RES as a research protocol that can benefit patients who require an urgent molecular diagnosis. Clinicians were encouraged to reach out to a representative of the study team (FSA) to propose cases any time of the day, any day of the week. The same representative judged the eligibility of cases to ensure consistency. Only cases for which an acceptable justification can be made that a rapid rather than routine molecular diagnosis is needed were enrolled. Eligible cases include (a) prenatal cases where a decision about pregnancy termination is required < 4 weeks before reaching the legal cutoff (120 days from conception, which is 18.5 weeks of gestation), (b) cases in intensive care units (usually neonatal or pediatric) where there is a lack of clarity about the diagnosis so that a confirmed diagnosis will either guide active management or lead to a de-escalation of management, and (c) cases where an urgent decision about eligibility for a transplant (liver or bone marrow transplant (BMT)) is needed. Informed consent was obtained from all participants prior to sampling, which typically involved venous blood collected in EDTA tbues. Occasionally, salivary samples were obtained, e.g., severe leukopenia. Amniotic fluid was used in prenatal cases. The study was approved by the local IRB (KFSHR RAC# 2,170,028).

### RES protocol

This was described in detail previously [2]. Briefly, rapid DNA extraction was performed using the PureLink Genomic DNA kit (Thermo Fisher, Carlsbad, CA, USA) as recommended by the manufacturer (https://www.thermofisher.com/document-connect/documentconnect.html?url=https://assets.thermofisher.com/TFS-Assets/LSG/manuals/purelink_genomic_man.pdf). Library preparation, emulsion PCR, enrichment, and sequencing were performed using the Ion Torrent AmpliSeq Whole Exome Sequencing protocol (https://www.thermofisher.com/sa/en/home/life-science/sequencing/dna-sequencing/exomesequencing/exome-sequencing-ion-torrent-next-generation-sequencing.html). Sequence alignment, indexing of the reference genome (hg19), variant calling, and annotation used a pipeline based on Burrows-Wheeler Aligner (BWA), Samtools, GATK (https://software.broadinstitute.org/gatk/), and Annovar, respectively. Essentially, variants were annotated using a combination of public knowledge databases available from the Annovar package and in-house databases which included collections of previously published Saudi disease-causing variants. Autozygosity analysis was included in the pipeline as previously described [2, 6]. Variant interpretation followed the ACMG guidelines [7]. The testing outcome was positive if a pathogenic/likely pathogenic variant was identified that explains the clinical indication in the appropriate zygosity. Variants of uncertain significance, including those with less than strong gene-disease assertion as per GenCC, were interpreted as ambiguous results (an exception was made when compound heterozygosity for a pathogenic variant and VUS were consistent with the phenotype in which case the case was labeled positive). All the remaining results were labeled negative. Given the known challenge of calling certain variant classes by the Ion Proton platform used in this study, we also ran all cases with “negative” and “ambiguous” results on Novaseq using the following protocol: Exons were captured and enriched using Illumina DNA Prep with Enrichment. Enrichment-bead-linked transposons (eBLT) were used to tagment 100–500 ng of gDNA and attach adapter sequences to the fragments. After eBLT clean-up, the addition of two indexes per sample by PCR amplification (5 cycles) was performed. Subsequently, individual libraries were pooled for a single hybridization reaction and capture. The last step consisted of a post-capture PCR amplification (8 cycles) prior to sequencing on a NovaSeq 6000 sequencer (Illumina, Inc., USA) as 150-bp paired-end reads, following the manufacturer’s protocols (Illumina, Inc., USA). The DNA sequence was mapped to and analyzed in comparison with the published human genome build (UCSC hg19 reference sequence) using a local installation of the Illumina DRAGEN Server v3 20040619 pipeline.

### Clinical utility

Clinicians were surveyed to request their input on the clinical utility of the result. Specifically, we used the same four domains developed by Dimmock et al. [4]:

- Category 1: major perceived specific changes in acute patient management or clinical outcome—These include screening for potential comorbidities associated with the genetic disease diagnosis, new sub-specialty consulted, changes in medications, changes in invasive procedures (including decisions regarding transplant and termination), changes in diet, changes in imaging studies, and changes in palliative care. Changes in clinical outcome were assessed by the successful use of targeted treatments, avoidance of complications, and institution of palliative care.
- Category 2: changes in communication—these include communication with families regarding outcomes, expectations, and prognosis.
- Category 3: changes in subsequent test ordering, i.e., triggering of additional confirmatory tests (testing for co-morbidities was not included here because it was part of category 1).
- Category 4: changes in other care (counseling, further monitoring, or research studies).

A yes answer to any of the above domains was recorded as “positive,” for the purpose of counting instances where a result had positive clinical utility.

### Economics of RES

RES in this study was based upon a single sample per Ion Torrent run. Reagent, consumable, and analytical costs were combined with overtime payments for the scientific and technical staff to calculate the overall cost of RES. Overhead costs and amortization of equipment were not included as these were not dedicated for RES and did not represent additional costs in our laboratory setting for this purpose.

The average annual healthcare cost for rare disease patients under 18 years of age for this study was adopted from the National Economic Burden of Rare Disease Study in the USA, undertaken by the EveryLife Foundation for rare diseases (https://everylifefoundation.org/burden-study). Institutional costs for liver or bone marrow transplantation were developed with Arthur D Little and Power Health in 2021 and adopted by our institution from the beginning of 2022, after analyzing our actual costs in the Charge Determination Master (comprehensive institutional database of items that could produce a charge) and bundle level costing developed with input from clinical areas.

## Results

### Cohort characteristics

The cohort comprises 189 unpublished cases (Additional file 1: Table S1). The age range was fetus to 40 years (exclusive of parents who underwent testing to deduce the cause of disease in a deceased child, see below), and the gender distribution was 91 females and 101 males. Intensive care units accounted for the majority of cases (53%). The justification for RES was (a) acute ICU management guidance (*n* = 67), (b) urgent decisions about transplant (liver or BMT) (*n* = 90), (c) prenatal decisions (*n* = 9), and others (*n* = 23). Additional file: Table S1 lists all the cases with their epidemiological and clinical characteristics.

### Platform limitations

Novaseq 6000 runs for cases declared negative on the Ion Torrent platform identified the following variants:

1. A pathogenic homozygous indel variant (*ERCC6L2*:NM_020207.4:c.3773_3774del: p.Met1258ThrfsTer29) in patient Pat-1056 with bone marrow failure
2. A pathogenic heterozygous indel variant (*SON*:NM_138927.4:c.3144del;p.Met1049Ter) in patient Pat-1108 with bilateral hydronephrosis, brain atrophy, hypotonia, dysmorphism, and congenital heart disease
3. A pathogenic homozygous deletion encompassing exons 1–5 of *FANCA* in patient Pat-1180 with suspected Fanconi anemia
4. A pathogenic homozygous startloss variant (*AIRE*:NM_000383.4:exon1:c.1A > G:p.?) in patient Pat-1163 with acute liver failure and autoimmune polyglandular syndrome
5. A pathogenic homozygous indel *HOXA1* variant (NM_005522.5:exon1:c.175dupG; p.Val59GlyfsTer119) in patient Pat-1186 with severe central hypoventilation

### Diagnostic yield

A positive finding was identified in 80 cases for a total of 82 variants (Table 1). These variants spanned 67 genes, 13 were novel, and 47 were predicted loss of function (LOF) (Additional file 1: Table S1). The overwhelming majority of positive results were autosomal recessive (69/80), and of these, all but 2 were homozygous (*n* = 67).

**Table 1:** Cases with positive molecular findings

**ID**	**Justification for RES**	**Clinical utility**	**Variant**	**OMIM-compatible diagnosis**
Pat-1139	Decision about liver transplant	Management (candidate for liver transplant), reproductive counseling (25% recurrence risk)	ABCB4:NM_000443:exon6:c.526C > T:p.Arg176Trp	Cholestasis, progressive familial intrahepatic 3
Pat-1000	Decision about termination	Management (prenatal testing), reproductive counseling (increase from 25% to 7/16, see text)	LMOD3:NM_198271:exon2:c.944_945del:p.Leu315GlnfsTer10	Nemaline myopathy 10
Pat-1037	Decision about management	Management (disease-specific treatment), reproductive counseling (50% recurrence risk)	HMBS:NM_001258208:exon12:c.706-2A > T	Porphyria, acute intermittent, nonerythroid variant
Pat-1117	Decision about management	Management (avoid steroid, list for transplant), reproductive counseling (25% recurrence risk)	PLCE1:NM_001165979:exon7:c.2134C > T:p.Gln712Ter	Nephrotic syndrome, type 3
Pat-1003	Decision about BMT	Management (candidate for BMT), reproductive counseling (25% recurrence risk)	ADA2:NM_177405.1:c.724_728del: p.Ser242ProfsTer5	Vasculitis, autoinflammation, immunodeficiency, hematologic defects syndrome
Pat-1039	Decision about BMT	Management (candidate for BMT), reproductive counseling (25% recurrence risk)	GALE:NM_001127621:exon3:c.151C > T:p.Arg51Trp	*GALE*-related thrombocytopenia (not yet listed in OMIM, see text)
Pat-1050	Decision about BMT	Management (candidate for BMT), reproductive counseling (25% recurrence risk)	MYSM1:NM_001085487:exon8:c.1168G > T:p.Glu390Ter	Bone marrow failure syndrome 4
Pat-1025	Decision about BMT	Management (candidate for BMT), reproductive counseling (25% recurrence risk)	MYSM1:NM_001085487:exon7:c.412C > T:p.R138Ter	Bone marrow failure syndrome 4
Pat-1001	Decision about termination	Prognostication	COL25A1:NM_001256074.2:c.1517del:p.Pro506HisfsTer25	*COL25A1*-related fetal akinesia (not yet listed in OMIM, see text)
Pat-1188	Decision about BMT	Management (candidate for BMT), reproductive counseling (25% recurrence risk)	RAG1:NM_000448:exon2:c.555delG:p.Lys186SerfsTer15	Severe combined immunodeficiency, B cell-negative
Pat-1098	Decision about management	Management (diagnosis-specific guidelines of drugs to avoid), reproductive counseling (50% recurrence risk in male offspring)	G6PD:NM_000402:exon6:c.653C > T:p.Ser218Phe	Anemia, nonspherocytic hemolytic, due to G6PD deficiency
Pat-1187	Decision about liver transplant	Management (candidate for liver transplant), reproductive counseling (25% recurrence risk)	ABCB4:NM_000443:exon13:c.1378A > T:p.Ile460Phe	Cholestasis, progressive familial intrahepatic 3
Pat-1006	Decision about liver transplant	Prognostication (progressive neurodegeneration), management (not candidate for liver transplant), reproductive counseling (25% recurrence risk)	SCYL1:NM_020680:exon10:c.1386 + 1G > T	Spinocerebellar ataxia, autosomal recessive 21
Pat-1028	Decision about BMT	Management (candidate for BMT), reproductive counseling (25% recurrence risk)	RAB27A:NM_004580:exon4:c.244C > T:p.Arg82Cys	Griscelli syndrome, type 2
Pat-1052	Decision about BMT	Management (rule out as a BMT donor, diagnosis-specific screening), reproductive counseling (7/16 recurrence risk)	BRCA2:NM_000059:exon19:c.8452G > T:p.Val2818Phe DCAF17:NM_001164821:exon4:c.436delC: p.Ala147HisfsTer9	Fanconi anemia, complementation group D1, Woodhouse-Sakati syndrome, dual diagnosis
Pat-1064	Decision about liver transplant	Management (not candidate for liver transplant), reproductive counseling (25% recurrence risk)	MPV17:NM_002437:exon4:c.279 + 1G > T	Mitochondrial DNA depletion syndrome 6 (hepatocerebral type)
Pat-1078	Decision about DNR	Management (palliative care), reproductive counseling (25% recurrence risk)	ACOX1: NM_007292:exon12:c.1728 + 1G > A	Peroxisomal acyl-CoA oxidase deficiency
Pat-1043	Decision about BMT	Management (candidate for BMT), reproductive counseling (25% recurrence risk)	JAK3: NM_000215.3:exon2:c.115dupC:p.Gln39ProfsTer13	SCID, autosomal recessive, T-negative/B-positive type
Pat-1042	Decision about management	Prognostication (life-long disease), management (avoid intestinal biopsy, plan lifelong TPN), reproductive counseling (25% recurrence risk)	EPCAM:NM_002354:exon5:c.499dupC:p.Gln167ProfsTer21	Diarrhea 5, with tufting enteropathy, congenital
Pat-1023	Decision about liver transplant	Management (candidate for liver transplant), reproductive counseling (25% recurrence risk)	ATP7B:NM_001005918:exon17:c.3574delC:p.Gln1192ArgfsTer6	Wilson disease
Pat-1038	Decision about liver transplant	Management (disease-specific treatment, not candidate for liver transplant), reproductive counseling (25% recurrence risk)	AKR1D1:NM_001190906:exon2:c.148C > T:p.Arg50Ter	Bile acid synthesis defect, congenital, 2
Pat-1045	Decision about DNR	Management (disease-directed investigation with urine organic acid and acylglycine profile showing severe ethylmalonic aciduria and elevated isobuturylglycine and isovalerylglycine), reproductive counseling (25% recurrence risk)	ETFB:NM_001014763:exon2:c.547C > T:p.Pro183Ser	Glutaric acidemia IIB
Pat-1047	Decision about management	Prognostication (once regression ensues, it is irreversible), management (palliative care), reproductive counseling (25% recurrence risk)	SLC25A42:NM_001321544:exon8:c.871A > G:p.Asn291Asp	Metabolic crises, recurrent, with variable encephalomyopathic features and neurologic regression
Pat-1053	Decision about DNR	Management (palliative care), reproductive counseling (only 25% probability of a healthy child)	FGFR3:NM_000142:exon9:c.1138G > A:p.Gly380Arg	Camptodactyly, tall stature, hearing loss syndrome; CATSHLS/autosomal recessive achondroplasia
Pat-1069	Decision about BMT	,Management (candidate for BMT), reproductive counseling (recurrence risk)	ELANE:NM_001972:exon5:c.640G > A:p.Gly214Arg	Neutropenia, severe congenital
Pat-1060	Decision about DNR	Management (not candidate for BMT), reproductive counseling (7/16 recurrence risk)	RAG1:NM_000448:exon2:c.554delG: p.Lys186SerfsTer15 TXNDC15:NM_001350735:exon3:c.499C > T:p.Arg167Trp	Severe combined immunodeficiency, B cell-negative
Pat-1054	Decision about BMT	Management (candidate for BMT), reproductive counseling (50% recurrence risk)	RTEL1:NM_001283009.2:c.3271_3273delGAC:p.Asp1091del	Dyskeratosis congenita
Pat-1058	Decision about DNR	Management (palliative care), reproductive counseling (25% recurrence risk)	OSTM1:NM_014028:exon2:c.415_416del:p.Gln140GlufsTer11	Osteopetrosis, autosomal recessive 5
Pat-1057	Decision about liver transplant	Management (not candidate for liver transplant, diagnosis-specific therapy), reproductive counseling (25% recurrence risk)	GALT:NM_001258332:exon6:c.364C > T:p.Arg122Cys	Galactosemia
Pat-1192	Decision about BMT	Management (not suitable as a BMT donor, diagnosis-specific screening), reproductive counseling (25% recurrence risk)	ATM:NM_000051:exon5:c.381delA:p.Val128Ter	Ataxia-telangiectasia
Pat-1067	Decision about BMT	Management (candidate for BMT), reproductive counseling (25% recurrence risk)	RAG2:NM_00536:exon2:c.110C > T:p.Pro37Leu	Severe combined immunodeficiency, B cell-negative
Pat-1074	Decision about BMT	Management (candidate for BMT), reproductive counseling (25% recurrence risk)	ZAP70:NM_001079:exon4:c.492delC p.His165ThrfsTer4	Immunodeficiency, 48
Pat-1065	Decision about management	Management (palliative care), reproductive counseling (25% recurrence risk)	PDHB:NM_000925:exon1:c.1A > G:p.?	Pyruvate dehydrogenase E1-beta deficiency
Pat-1062	Decision about DNR	Management (diagnosis-specific treatment), reproductive counseling (25% recurrence risk)	LIPA:NM_000235:exon4:c.260G > T:p.Gly87Val	Wolman disease
Pat-1099	Decision about BMT	Management (not candidate for BMT), reproductive counseling (25% recurrence risk)	OSTM1:NM_014028:exon2:c.415_416del: p.Gln140GlufsTer11	Osteopetrosis, autosomal recessive 5
Pat-1076	Decision about management	Management (diagnosis-specific treatment), reproductive counseling (25% recurrence risk)	KCNJ11:NM_000525:exon1:c.112A > G:p.Lys38Glu	Hyperinsulinemic hypoglycemia, familial, AR
Pat-1072	Decision about liver transplant	Management (not a candidate for liver transplant), reproductive counseling (25% recurrence risk)	ANKS6:NM_173551:exon11:c.2142G > T:p.Lys714Asn	Nephronophthisis, 16
Pat-1085	Decision about management	Management (diagnosis-specific screening and prophylaxis), reproductive counseling (25% recurrence risk)	C8B:NM_000066:exon9:c.1282C > T:p.Arg428Ter	C8 deficiency, type II
Pat-1184	Decision about management	Management (diagnosis-specific treatment), reproductive counseling (25% recurrence risk)	CYP19A1:NM_001347256.2:exon3:c.343C > T:p.Arg115Ter	Aromatase deficiency
Pat-1101	Decision about liver transplant	Management (candidate for liver transplant), reproductive counseling (25% recurrence risk)	ABCB4:NM_000443:exon23:c.2906G > A:p.Arg969His	Cholestasis, progressive familial intrahepatic 3
Pat-1087	Decision about BMT	Management (candidate for BMT), reproductive counseling (25% recurrence risk)	AK2:NM_001319142:exon5:c.398G > C:p.Arg133Pro	Reticular dysgenesis
Pat-1104	Decision about DNR	Management (palliative care), reproductive counseling (minimal recurrence risk in parents)	ACTA1:NM_001100:exon4:c.593G > A:p.Arg198His	Myopathy, actin, congenital, with cores
Pat-1094	Decision about BMT	Management (candidate for BMT), reproductive counseling (25% recurrence risk)	JAK3:NM_000215:exon3:c.307C > T:p.Arg103Cys and JAK3:NM_000215:exon6:c.678_679del:p.Cys227ProfsTer49	SCID, autosomal recessive, T-negative/B-positive type
Pat-1106	Decision about management	Management (diagnosis-specific therapy), reproductive counseling (25% recurrence risk)	SLC25A15:NM_014252.4:c.-69-41_55 + 58del	Hyperornithinemia-hyperammonemia-homocitrullinemia syndrome
Pat-1110	Decision about BMT	Management (candidate for BMT), reproductive counseling (25% recurrence risk)	STXBP2:NM_001127396:exon16:c.1421C > T:p.Pro474Leu	Hemophagocytic lymphohistiocytosis, familial, 5, with or without microvillus inclusion disease
Pat-1141	Decision about liver transplant	Management (not candidate for liver transplant), reproductive counseling (25% recurrence risk)	MPV17:NM_002437:exon4:c.279 + 1G > T	Mitochondrial DNA depletion syndrome 6 (hepatocerebral type)
Pat-1113	Decision about liver transplant	Management (not candidate for liver transplant), reproductive counseling (25% recurrence risk)	MPV17:NM_002437:exon4:c.279 + 1G > T	Mitochondrial DNA depletion syndrome 6 (hepatocerebral type)
Pat-1114	Decision about liver transplant	Management (candidate for liver transplant), reproductive counseling (25% recurrence risk)	ATP7B:NM_001005918:exon17:c.3574delC:p.Gln1192ArgfsTer6	Wilson disease
Pat-1116	Decision about liver transplant	Management (candidate for liver transplant), reproductive counseling (25% recurrence risk)	AIRE:NM_000383:exon10:c.1192delC:p.Pro398ArgfsTer82	Autoimmune polyendocrinopathy syndrome, type I, with or without reversible metaphyseal dysplasia
Pat-1119	Decision about management	Management (avoid diazoxide, good candidate for pancreatectomy), reproductive counseling (25% recurrence risk)	KCNJ11:NM_000525:exon1:c.101G > A:p.Arg34His	Hyperinsulinemic hypoglycemia, familial, AR
Pat-1120	Decision about DNR	Management (palliative care), reproductive counseling (25% recurrence risk)	PC:NM_022172:exon6:c.736G > A:p.Glu246Lys and PC:NM_022172:exon11:c.1486C > T:p.Arg496Trp	Pyruvate carboxylase deficiency
Pat-1131	Decision about BMT	Management (candidate for BMT), reproductive counseling (25% recurrence risk)	PNP:NM_000270:exon2:c.12-1G > C	Immunodeficiency due to purine nucleoside phosphorylase deficiency
Pat-1143	Decision about BMT	Management (candidate for BMT), reproductive counseling (25% recurrence risk)	RAB27A:NM_183234.2:c.467 + 1_467 + 4del	Griscelli syndrome, type 2
Pat-1142	Decision about management	Management (diagnosis-specific treatment), reproductive counseling (25% recurrence risk)	MTHFR:NM_001330358:exon7:c.1252C > T:p.Arg418Cys	Homocystinuria
Pat-1138	Decision about management	None	NIPBL:NM_015384:exon10:c.3060_3063del:p.Glu1021ThrfsTer22	Cornelia de Lange syndrome 1
Pat-1156	Decision about BMT	Management (candidate for BMT), reproductive counseling (25% recurrence risk)	CYBA:NM_000101.4:exon4:c.261C > A: p.Tyr87Ter	Chronic granulomatous disease 4, autosomal recessive
Pat-1150	Decision about management	Management (diagnosis-specific management), reproductive counseling (25% recurrence risk)	PTF1A:NM_178161.3:exon1:c.571C > A:p.Pro191Thr	Pancreatic and cerebellar agenesis
Pat-1083	Decision about BMT	Management (candidate for BMT), reproductive counseling (25% recurrence risk)	RAG1:NM_000448.3:exon2:c.2210G > A:p.Arg737His	Severe combined immunodeficiency, B cell-negative
Pat-1153	Decision about management	Management (diagnosis-specific treatment), reproductive counseling (25% recurrence risk)	SCNN1A:NM_001159575.1:exon4:c.944 + 1G > A	Pseudohypoaldosteronism, type I
Pat-1152	Decision about management	Management (diagnosis-specific treatment), reproductive counseling (25% recurrence risk)	ABCC8:NM_000352.6:exon4:c.563A > G:p.Asn188Ser	Diabetes mellitus, permanent neonatal 3
Pat-1154	Decision about liver transplant	Management (diagnosis-specific treatment), reproductive counseling (25% recurrence risk)	AKR1D1:NM_001190906.2:exon8:c.817 T > C:p.Trp273Arg	Bile acid synthesis defect, congenital, 2
Pat-1159	Decision about management	Management (diagnosis-specific treatment), reproductive counseling (50% recurrence risk)	RB1:NM_000321.3:c.1450_1451del:p.Met484ValfsTer8	Retinoblastoma
Pat-1157	Decision about BMT	Management (not candidate for BMT), reproductive counseling (25% recurrence risk)	OSTM1:NM_014028.4:exon2:c.415_416del: p.Gln140GlufsTer11	Osteopetrosis, autosomal recessive 5
Pat-1158	Decision about BMT	Management (candidate for BMT), reproductive counseling (25% recurrence risk)	RAG2:NM_000536.4:exon2:c.686G > C:p.Arg229Pro	Severe combined immunodeficiency, B cell-negative
Pat-1160	Decision about BMT	Reproductive counseling (25% recurrence risk)	GNS:NM_002076.4:exon6:c.732C > A: p.Tyr244Ter	Mucopolysaccharidosis type IIID
Pat-1002	Decision about management	Management (candidate for BMT), reproductive counseling (25% recurrence risk)	RFXANK:NM_134440.2:c.268 + 1G > C	Bare lymphocyte syndrome, type II, complementation group B
Pat-1090	Decision about BMT	Reproductive counseling (25% recurrence risk)	DIAPH1:NM_001079812:exon15:c.2305C > T: p.Gln769Ter	Seizures, cortical blindness, microcephaly syndrome
Pat-1029	Decision about management	Management (candidate for BMT), reproductive counseling (25% recurrence risk)	SMARCD2:NM_001098426:exon11:c.1429C > T:p.Arg477Ter	Specific granule deficiency 2
Pat-1011	Decision about management	Management (diagnosis-specific screening), reproductive counseling (50% recurrence risk)	TTN:NM_003319:exon166:c.64806G > A: p.Trp21602Ter	Cardiomyopathy, dilated, 1G
Pat-1013	Decision about management	Management (candidate for BMT), recurrence risk (50% recurrence risk for male offspring)	SH2D1A:NM_001114937:exon1:c.23A > C:p.His8Pro	Lymphoproliferative syndrome, X-linked, 1
Pat-1075	Decision about DNR	Management (palliative care), reproductive counseling (25% recurrence risk)	PEX5:NM_001351132.2:c.1578 T > G;p.Asn526Lys	Peroxisome biogenesis disorder, 2A (Zellweger)
Pat-1121	Decision about management	Reproductive counseling (25% recurrence risk)	PRF1:NM_001083116:exon3:c.880delC: p.Gln294LysfsTer36	Hemophagocytic lymphohistiocytosis, familial, 2
Pat-1146	Decision about management	Management (avoid selumetinib, diagnosis-specific surveillance), reproductive counseling (50% recurrence risk)	SMARCB1:NM_001317946:exon8:c.1145 + 2 T > C	Schwannomatosis
Pat-1145	Decision about management	Management (diagnosis-specific treatment), reproductive counseling (25% recurrence risk)	MEFV:NM000243:exon10:c.2082G > A:p.Met694Ile	Familial Mediterranean fever
Pat-1151	Decision about management	Prognostication (natural history), reproductive counseling (25% recurrence risk)	ISCA2:NM_194279.4:exon3:c.229G > A:p.Gly77Ser	Multiple mitochondrial dysfunctions syndrome 4
Pat-1161	Decision about management	Management (candidate for BMT), reproductive counseling (50% recurrence risk in male offspring)	FOXP3:NM_001114377.2:exon11:c.1085G > A:p.Arg362Gln	Immunodysregulation, polyendocrinopathy, enteropathy, X-linked
Pat-1140	Decision about liver transplant	Management (diagnosis-specific treatment), reproductive counseling (25% recurrence risk)	ATP7B:NM_001330579:exon6:c.1978 T > C:p.Ser660Pro	Wilson disease
Pat-1021	Decision about termination	Management (prenatal testing), reproductive counseling (recurrence risk)	ZDHHC15:NM_001146256:exon4:c.421A > G:p.Met141Val and TYR:NM_000372:exon4:c.1322delC:p.Ser	Albinism oculocutaneous
Pat-1088	Decision about BMT	Management (diagnosis-specific treatment), reproductive counseling (25% recurrence risk)	TCN2:NM_001184726:exon1:c.64 + 4A > T	Transcobalamin II deficiency
Pat-1132	Decision about DNR	Management (diagnosis-specific treatment), reproductive counseling (25% recurrence risk)	SLC52A3:NM_033409:exon2:c.211G > A:p.Glu71Lys	Brown-Vialetto-Van Laere syndrome 1

We highlight below some interesting clinical diagnoses revealed by RES.

#### Support of previously reported novel gene-disease assertions

Spinocerebellar ataxia, autosomal recessive 21 is the only disease listed in OMIM under *SCYL1*. However, Lenz et al. reported a novel *SCYL1*-related phenotype known as CALFAN (low γ-glutamyl-transferase cholestasis, acute liver failure, and neurodegeneration) syndrome [8]. Patient Pat-1006 who presented with acute liver failure and encephalopathy was found to have a homozygous splicing variant *SCYL1*:NM_020680:exon10:c.1386 + 1G > T, which supports this association.

*GALE* encodes UDP-galactose-4-prime-epimerase, and its deficiency is known to cause galactosemia. Surprisingly, an Arab founder variant (NM_001127621:exon3:c.151C > T: p.Arg51Trp) has been shown to cause a distinct phenotype limited to thrombocytopenia [9]. Patient Pat-1039 with thrombocytopenia, immune hemolytic anemia, low IgA and IgM, and recurrent infections was enrolled for RES because an urgent decision was needed for bone marrow transplant. He was found to be homozygous for the same founder variant. Thus, his phenotype both corroborates and expands this distinct *GALE*-related phenotype.

#### Expansion of previously reported phenotypes

Pat-1001 is a fetus with fetal akinesia who was found on RES to be homozygous for a LOF variant in *COL25A1* (NM_001256074.2:c.1517del:p.Pro506HisfsTer25). Only an eye-limited phenotype is listed in OMIM under *COL25A1* (Fibrosis of extraocular muscles, congenital, 5). During the preparation of this manuscript, we became aware of the additional cases of *COL25A1*-related fetal akinesia with generalized muscle involvement (revision submitted). Thus, this represents a clinically significant phenotypic expansion.

#### Revelation of previously unsuspected diagnoses

While the specific diagnosis was not suspected in the majority of cases, some remarkable examples are worth highlighting. Patient Pat-1192 is a 7-year-old child who was being considered as a candidate for an urgent bone marrow transplant of a sibling with aplastic anemia. RES was performed on both siblings to diagnose the cause of aplastic anemia in the index, so it can be ruled out in the sibling (potential donor). Surprisingly, while the cause of aplastic anemia was not identified, the sibling Pat-1055 was found to be homozygous for a pathogenic variant in *ATM* thus establishing a clinically unsuspected diagnosis of ataxia telangiectasia syndrome and ruling out the possibility of serving as a donor. Similarly, patient Pat-1098 with a cyanotic heart disease was having repeated hemolytic crises with no clear etiology. The finding of a pathogenic *G6PD* variant was surprising because prior tests revealed normal G6PD levels. It was later found that the levels were measured too soon after blood transfusion to truthfully reflect the endogenous enzyme activity. Another example is patient Pat-1160 who presented with features of hemophagocytic lymphohistiocytosis (HLH) and a history of a sibling who died in infancy with a similar presentation. Unexpectedly, this patient was found to have a homozygous pathogenic variant in *GNS*. It remains unclear how this finding is related, if at all, to the main presentation.

### Clinical utility of RES

**Fig. 1:**
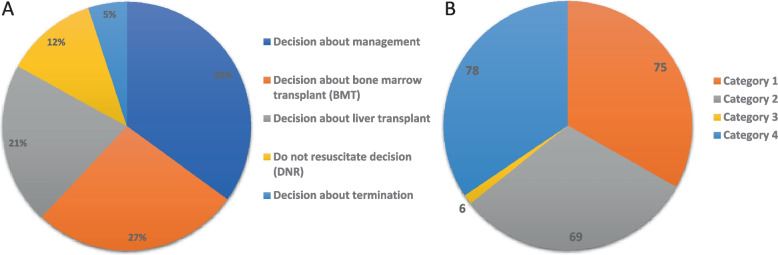
Summary of clinical utility (**A**) and clinical indications (**B**). Categories correspond to the ones listed in the text. Please note there is an overlap in the cases and corresponding categories because multiple facets of clinical utility were encountered in some patients

The clinical team identified one or more aspects of clinical utility in the overwhelming majority of cases that received positive findings (79/80) (Fig. 1). These can be classified as follows (Table 2, Additional file 2: Fig. S1).

**Table 2:** Number of patients with demonstrable clinical utility (four domains)

**Changes in acute patient management or clinical outcome**	***n* = 75**^**a**^
Changes in medications	11
Changes in invasive procedures	16
Changes in clinical outcome	23
Decision about palliative care	13
Decision about transplant	30
Changes in diet	3
Changes in imaging studies	1
**Changes in communication**	***n* = 69**^**a**^
Communicate a specific diagnostic label not suspected prior to flash ES	66
Explains the natural history of disease	67
**Changes in subsequent test ordering**	***n* = 6**
**Changes in other care**	***n* = 78**^**a**^
Counseling	74
Further monitoring	10

^a^Please note there is an overlap in the cases and corresponding categories

#### Major perceived specific changes in acute patient management

**Fig. 2:**
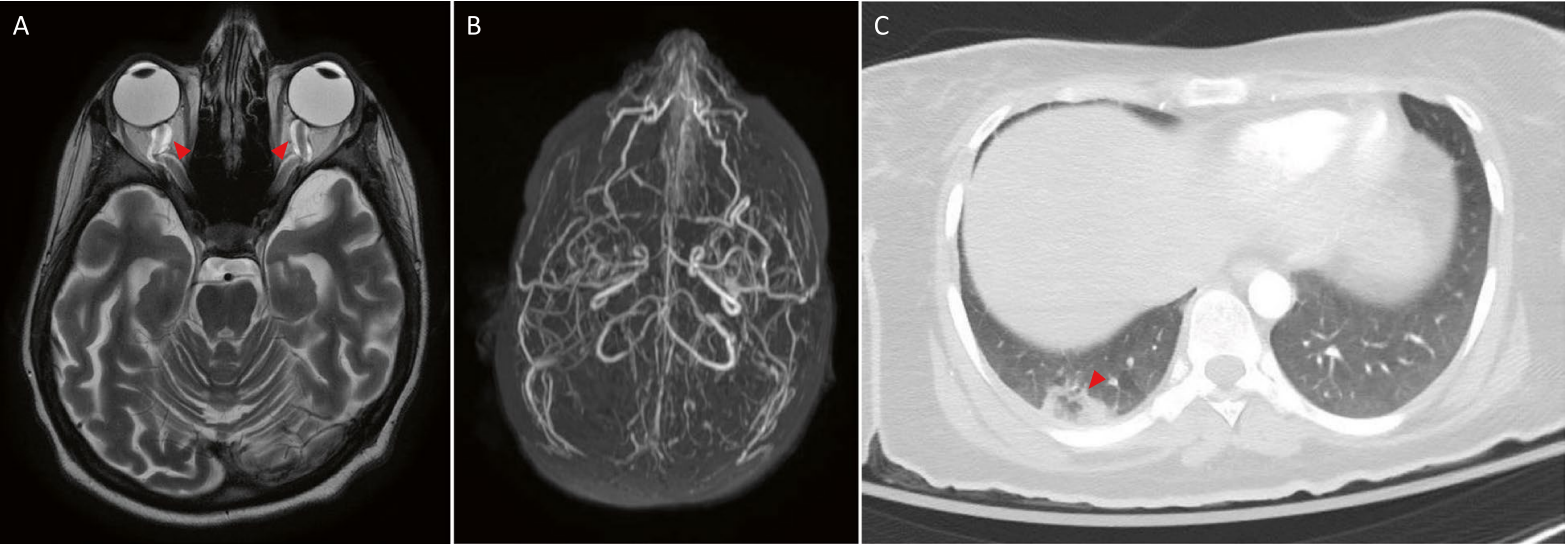
An illustrative case Pat-1142 of the clinical utility of RES. MRI of the brain showed brain atrophy, with tortuous and prominent CSF spaces even in the optic nerves bilaterally (**A**). MR venography revealed extensive thrombosis of the cerebral sinuses (**B**). CT chest revealed extensive bilateral pulmonary emboli with right lower lobe pulmonary infarction evident by the reversed halo sign (**C**)

In the majority of positive cases (94%), patients received significant changes in their clinical management. RES informed an urgent decision regarding liver transplant in 15 cases (7 deemed eligible and 8 ineligible) and BMT in 26 cases (21 deemed eligible and 5 ineligible). Additional diagnosis-specific management plans were enabled in 29 cases. Some of the remarkable examples include patient Pat-1057 that presented with acute liver failure and had complete resolution of symptoms after the institution of galactosemia-specific dietary management (diagnosis was unfortunately missed by newborn screening). Patient Pat-1088 who presented with pancytopenia, myelodysplastic syndrome on bone marrow biopsy, 5% blasts in the peripheral blood, mild developmental delay, abnormal skin pigmentation, and intestinal obstructions had a dramatic response to hydroxycobalamin injections after he was found to have a cobalamin defect caused by a homozygous *TCN2* variant. Ornithine supplementation and protein restriction led to complete clinical recovery in patient Pat-1106 whose hyperammonemic encephalopathy was found to be due to *SLC25A15*-related hyperornithinemia-hyperammonemia-homocitrullinemia syndrome. The PICU team almost placed patient Pat-1132 on do not resuscitate (DNR) status after discussing with his parents the grim prognosis of his progressive muscle weakness, bulbar palsy, and respiratory failure. However, the prompt administration of riboflavin upon the identification of *SLC52A3*-related riboflavin transporter defect led to a quick and dramatic clinical improvement, and the patient is now followed on an outpatient basis. Please note that although the *SLC52A3* and *TCN2* variants are VUS, we opted to include these cases because of a recent publication advocating the use of the expected response to therapy to support the pathogenicity of VUS in the respective genes [10]. Similarly, clinical improvement was notable in patient Pat-1142 who presented with rapid unexplained cognitive decline and evidence of extensive vessel disease in the brain (with associated atrophy) and body (extensive thrombosis) after her diagnosis of *MTHFR*-related homocystinuria diagnosis prompted appropriate therapy (Fig. 2). We also highlight the instances where the molecular diagnosis led to the cessation of ineffective therapies. For example, steroids and immunosuppressants were discontinued in patient Pat-1117 with *PLCE1*-related nephrotic syndrome, and the patient was enlisted for renal transplant instead. The diagnosis of *KCNJ11*-related congenital hyperinsulinism in patient Pat-1119 led to the cessation of diazoxide and pursuit of pancreatectomy instead with excellent results. In another example, the managing team was able to avoid the use of selumetinib when RES revealed the cause of schwannomatosis in patient Pat-1146 as neurilemmomatosis rather than *NF1*-related neurofibromatosis.

#### Changes in communication within healthcare teams and with families

In the majority of cases (83%), the clinicians appreciated the opportunity to communicate a specific diagnostic label not suspected prior to RES and explain the natural history of the disease to provide informed prognostication (Additional file 1: Table S1).

#### Changes in subsequent test ordering

The molecular diagnosis triggered very few instances (8%) of confirmatory tests (screening for comorbidities and other features of the disease were not included, see above) (Additional file 1: Table S1).

#### Changes in other care (counseling, further monitoring, or research studies)

The autosomal recessive nature of the causal variant in the overwhelming majority of positive cases (69/80, 86%) enabled reproductive counseling. Parents were offered options for preimplantation genetic testing or prenatal diagnosis. The 25% recurrence risk was complicated in a few instances where multiple pathogenic variants were identified. For example, patient Pat-1000 sought counseling during pregnancy for a pathogenic *FARS2* variant identified in a deceased child, but another deceased child had a different phenotype (severe hypotonia) and was found heterozygous for the familial *FARS2* variant. RES was requested on the couple and a stored DNA sample from the other deceased child. This revealed homozygosity for *LMOD3*:NM_198271:exon2:c.944_945del: p.Leu315GlnfsTer10, which confirmed the diagnosis of nemaline myopathy. Thus, the recurrence risk was adjusted from 1/4 to 7/16, and prenatal diagnosis for both variants was offered. Reproductive counseling was also offered for X-linked conditions. Parental testing was requested in the case of autosomal dominant variants and where de novo status was confirmed, a minimal residual risk was offered, and the possibility of parental mosaicism was discussed.

Of 189 cases analyzed in this study using RES, only one case of multilocus pathogenicity was observed in a patient (Pat-1052) who was homozygous for two pathogenic variants: *BRCA2*:NM_000059:exon19:c.8452G > T:p.Val2818Phe and *DCAF17*:NM_001164821:exon4:c.436delC: p.Ala147HisfsTer9.  Although patients Pat-1060 and Pat-1021 also had more than one gene involved, we did not include them because we only counted cases where all variants can be classified as at least likely pathogenic.

### Secondary findings

Consistent with our previous experience [2, 6], the percentage of cases with ACMG secondary findings was small (*n* = 2), and the findings are listed in Additional file 1: Table S1.

### Cost reduction and net saving by RES

The RES cost per sample in our study was comfortably ascertained to be USD 2000. At this price, the total diagnostic cost for the 189 cases described is USD 378,000. The average annual healthcare cost for rare disease patients under 18 years of age is USD 80,436 (https://everylifefoundation.org/burden-study). In our study, the average age of patients accepted was approximately 3 years. The annual cost of treatment for these 189 patients would be estimated at USD 15,202,404. The diagnostic cost of USD 378,000 represents 2.48% of this annual cost. In particular, 26 of the positive cases in this study justified proceeding with bone marrow transplantation (institutional cost of USD 320,000) or liver transplantation (institutional cost of USD 261,000) with a total procedural cost of USD 14,628,000. The RES diagnostic cost for all cases in this study represents 1.27% of the estimated annual treatment and/or procedural cost of USD 29,830,404. RES results facilitated reproductive counseling in 80 cases each with the potential to prevent one or more future live births of affected babies. On the conservative assumption that these 80 cases would be associated with 1 future sibling with a 25% risk of being affected, the potential to prevent such birth would generate an annual saving in healthcare costs of USD 1,608,720 multiplied by the average annual lifespan. Similarly, RES results changed the management of 9 patients in which expensive and contraindicated transplant procedures (6 liver and 3 bone marrow transplants) were avoided with savings of USD 2,526,000. In addition, RES results changed the active management of 3 patients in ICU to palliative care and do not resuscitate status, cost savings of USD 198,670 assuming an on average 30-day stay in ICU for these patients. RES from this cohort alone was able to generate healthcare cost savings of more than USD 4,333,390 representing a net saving of at least USD 3,955,390.

## Discussion

Since its first introduction in 2012 [5], RGS/RES has received a growing interest in view of its potential to bring precision medicine a step closer to the point-of-care with a high diagnostic yield. It has been shown to reduce cost [4, 11], and improve resource utilization [12], and it scores favorably on parental satisfaction [13, 14]. Importantly, its clinical utility has been demonstrated by multiple groups, typically in the neonatal and pediatric ICU settings [4, 11, 12, 15–18]. The latter is key because it was only after demonstrating clinical validity that traditional ES was endorsed as a standard practice eligible for coverage even though its high diagnostic yield had been evident since the early stages. Similarly, robust data on the clinical utility of RGS/RES will be needed to justify the coverage of this specialized workflow that tends to cost more than traditional ES. These data should include different settings, including inbred populations which are largely lacking in clinical validity studies of RGS/RES [19].

There are several other factors that make the analysis of RES/RGS clinical validity in inbred populations important. The autozygosity in these populations makes it likely to encounter extremely rare, even novel, indications of RES/RGS. This is readily seen in our study where several diagnoses are so rarely reported they are not yet listed in OMIM. The predominance of autosomal recessive diseases in our highly consanguineous population also presents an opportunity to test the clinical utility of RES/RGS under unique circumstances. For example, the ability to deduce the cause of death in a previous child by searching for the shared carrier status of a lethal variant in consanguineous couples (molecular autopsy by proxy [20, 21]) and to utilize this variant for prenatal diagnosis is a form of clinical utility that is hard to appreciate in other settings. Another example is reproductive counseling, which has a different utility in the case of autosomal recessive diseases compared to de novo dominant variants in outbred populations. Indeed, we have previously shown that > 90% of families with autosomal recessive diseases in our population make active reproductive decisions, e.g., prenatal diagnosis or preimplantation genetic testing, after counseling [22].

The clinical utility of RES needs also to be considered in the context of the sequencing technology utilized. Ion Torrent RES offered flexibility and economic advantages related to single sample runs and speed. However, the Ion Torrent exomes used in this study demonstrated reduced sensitivity for indels, particularly in homopolymeric regions, as evidenced by false negatives relative to subsequent confirmatory exomes run using a Novaseq platform. This, while affecting a small number of cases, is nevertheless a serious limitation of Ion Torrent sequencing that should be considered when applied in this setting.

From an economic perspective, annual healthcare costs and/or procedural (liver or bone marrow transplant) cost alone for our patient cohort more than justify the application of RES which contributed very fractionally (1.27%) to overall costs. However, in the context of lifetime healthcare costs associated with the treatment of patients with rare diseases, the cost of RES is relatively further diminished, with justification of the test and clinical value being significantly amplified. Net savings in healthcare costs of applying RES in this study cohort represented a > 10.46 multiplier relative to RES cost.

## Conclusions

This study shows a high clinical utility of RES in our consanguineous population using a comparable definition to previous studies in outbred populations. Additionally, we highlight several unique facets of clinical utility in this special setting. We hope our results will add to the growing body of evidence supporting the deployment of RES as a standard clinical test for acute indications.

## Supplementary Information

**Additional file 1: Table S1.** Detailed clinical and molecular data of the study cohort.xls

**Additional file 2.** Supplementary figure.

## Data Availability

All variants’ data has been deposited to ClinVar (https://www.ncbi.nlm.nih.gov/clinvar/) and ClinVar accession IDs are provided in Additional file 1: Table S1. Due to local IRB (King Faisal Specialist Hospital and Research Center Research Advisory Council) regulations to protect the privacy of human subjects, individual-level raw data cannot be deposited in public databases.
