# Supplementary material for: The clinical utility of rapid exome sequencing in a consanguineous population
Source: Genome Med. 2023 Jun 21;15:44. doi: 10.1186/s13073-023-01192-5 (PMC10283251; doi:10.1186/s13073-023-01192-5)
Supplement: Supplementary file 2 — Additional file 2. Supplementary figure. [file 13073_2023_1192_MOESM2_ESM.pptx]

## Slide 1
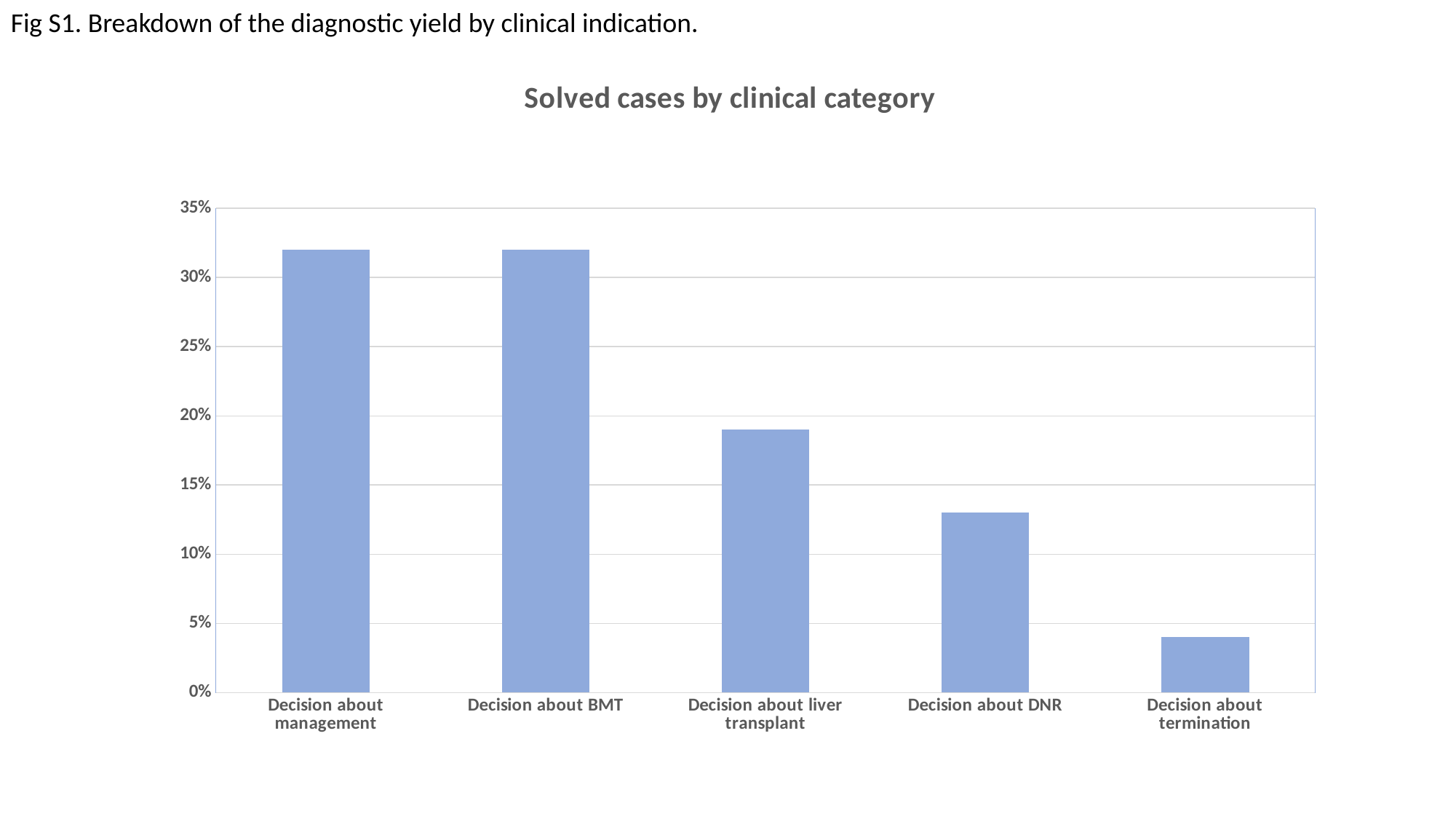

Fig S1. Breakdown of the diagnostic yield by clinical indication.
### Chart: Solved cases by clinical category
| Category | Solved cases |
|---|---|
| Decision about management | 0.32 |
| Decision about BMT | 0.32 |
| Decision about liver transplant | 0.19 |
| Decision about DNR | 0.13 |
| Decision about termination | 0.04 |
